# Scapholunate Advanced Collapse (SLAC) and Scaphoid Nonunion Advanced Collapse (SNAC): A Review of Treatment Options for Stage II

**DOI:** 10.7759/cureus.59014

**Published:** 2024-04-25

**Authors:** Spyridon Maris, Emmanouil Apergis, Alexandros Apostolopoulos, Dimitra Melissaridou, Panagiotis Koulouvaris, Panayiotis J Papagelopoulos, Olga Savvidou

**Affiliations:** 1 Department of Orthopaedics and Traumatology, General Hospital Hellenic Red Cross Korgialenio Benakio, Athens, GRC; 2 Department of Orthopaedics, General Hospital Hellenic Red Cross Korgialenio Benakio, Athens, GRC; 3 Department of Orthopaedics, East Surrey Hospital, Surrey and Sussex Healthcare NHS Trust, Redhill, GBR; 4 First Department of Orthopaedic Surgery, Attikon University General Hospital, Athens, GRC; 5 First Department of Orthopaedic Surgery, National and Kapodistrian University of Athens School of Medicine, Athens, GRC

**Keywords:** wrist arthrodesis, wrist tenodesis, wrist arthritis, snac ii, slac ii

## Abstract

Scapholunate advanced collapse (SLAC) and scaphoid nonunion advanced collapse (SNAC) represent clinical entities identified by a pattern of predictable degenerative changes. They are the most common causes of wrist arthritis.

Both entities can remain asymptomatic for many years and may go undiagnosed. Diagnosis is usually confirmed through clinical examination, which reveals progressive wrist pain and instability. Radiographically, degenerative changes in the radiocarpal and midcarpal joints are present, as well as nonunion of the scaphoid fracture in SNAC.

The management differs according to the stage. Particularly in this review article, we reviewed the treatment options for stage II SLAC and SNAC wrist. In addition to the well-described surgical techniques such as proximal row carpectomy and four-corner fusion, alternatives such as capitolunate arthrodesis, three-corner fusion, and soft tissue procedures like capsulodesis and tenodesis are available.

Proximal row carpectomy and partial arthrodeses yield comparable results. Soft tissue procedures are viable alternatives and are preferred in younger patients to avoid early salvage operations.

## Introduction and background

Scapholunate advanced collapse (SLAC) represents the most common cause of wrist osteoarthritis and accounts for 72% of wrist osteoarthritis cases [1]. Watson HK and Ballet FL first described SLAC lesions in 1984. They reviewed 4000 wrist x-rays and concluded that arthritis sequentially developed between the scaphoid, lunate, and radius, representing 57% of cases [1]. They concluded that incompetence of the scapholunate ligament led to a sequence of degenerative changes in the radioscaphoid junction in the early stages (I and II) and later to the midcarpal joint (stage III). In Watson HK and Ballet FL's original descriptions, the classification of SLAC wrist had three stages, without a pancarpal stage 4, as has occasionally been described, since the spherical radiolunate articulation was spared [2].

Vender MI et al. [3] originally described scaphoid nonunion advanced collapse (SNAC) in 1987. It is a similar clinical entity to SLAC since it is identified by a series of predictable degenerative changes. However, the initial injury in SNAC is the fracture and nonunion of the scaphoid, unlike in SLAC, where the injury of the SL ligament is the cause. SNAC lesions can also be caused by scaphoid malunion [4].

Both lesions alter carpal kinematics and progress to wrist arthritis in a predictable pattern. These entities are diagnosed clinically and mainly with imaging methods, including radiographs, CT, and MRI, which help diagnose SLAC and monitor its progression [5].

There are different treatment options depending on the stage of the disease. Asymptomatic wrists do not require treatment [5]. Symptomatic SLAC/SNAC wrists can be treated with radial styloidectomy, denervation, proximal row carpectomy (PRC), partial (4-corner fusion, capitolunate fusion), or complete wrist arthrodesis [6]. An additional treatment option for SNAC wrists is the distal “ununited” scaphoid excision [6].

## Review

Scapholunate interosseous ligament (SLIL)

The SLIL is the primary stabilizer between the scaphoid and lunate. Secondary scapholunate stabilizers include the scaphotrapezium (STT complex), the radioscaphocapitate, scaphocapitate, long and short radiolunate ligaments volarly, and dorsally, the dorsal intercarpal (with dorsal capsuloscapholunate septum) and dorsal radiocarpal ligaments [5].

**Figure 1:**
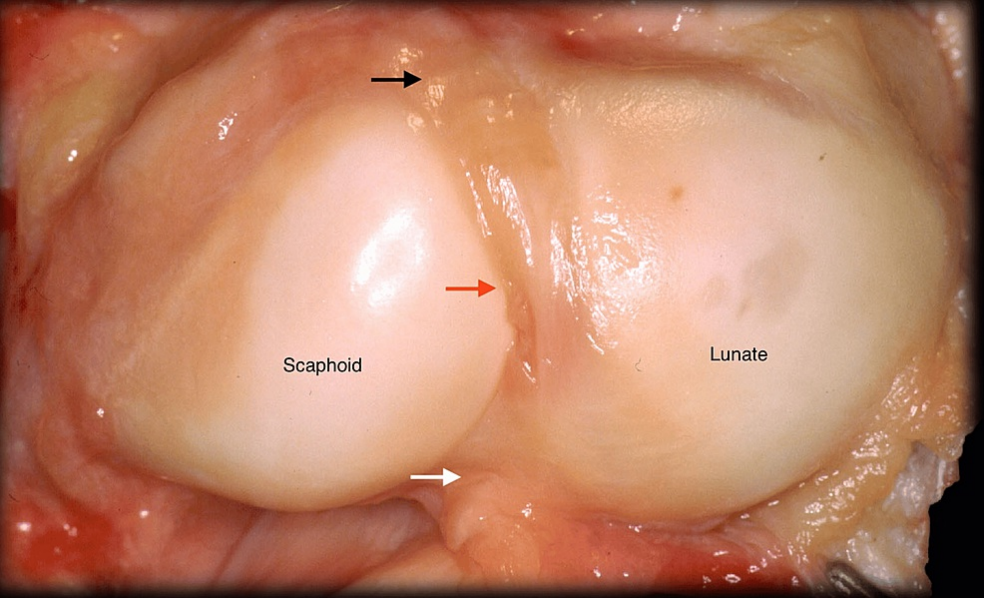
Scapholunate ligament as viewed from the radiocarpal joint. Black arrow: Dorsal part; White arrow: Volar part; Red arrow: Membranous part. Source: Dr. Emmanouil Apergis

The scapholunate ligament comprises three parts (Figure 1): the dorsal, membranous, and volar components. The dorsal and volar regions histologically mimic capsular ligaments, while the membranous part is composed of fibrocartilage with few collagen fascicles.

The dorsal part is the thickest and strongest; it has a trapezoidal shape and is composed of transversely oriented collagen bundles surrounded by connective tissue. It plays the most crucial role in stabilization. It measures approximately 2-4 mm in thickness and 5 mm in width. The distal part of the dorsal band attaches to the scaphotriquetral ligament.

The membranous part is composed of fibrocartilage and does not contain nerves or vessels. It is approximately 1 mm thick, 4 mm long, and 11 mm wide.

The volar part measures approximately 1-2 mm in thickness, 3-5 mm in length, and 4-7 mm in width. This part is not visible because the long radiolunate ligament covers its palmar surface.

The SLIL is a strong structure that requires up to 300N of distraction force to rupture. Most of the distraction strength is found in the dorsal region. The volar part ruptures with 150N of stress, and the membranous part with 25-50N of stress [7].

With arthroscopy, our understanding of the scapholunate joint has gradually evolved. For example, it was discovered that scapholunate instability could exist without ligament tears or radiographic diastasis [8-10]. Furthermore, the scaphoid can be destabilized and dissociated from the lunate by damaging at least one extrinsic stabilizer [10-12]. The SLIL is insufficient to stabilize the scaphoid and lunate and should no longer be considered the sole stabilizer of the joint [13].

Etiology 

SLAC wrist results from an SLIL lesion, which can be either traumatic or non-traumatic. Non-traumatic causes of SLAC wrist include rheumatoid arthritis, calcium pyrophosphate dehydrate deposition disease (CPPD), neuropathic diseases, Kienböck's disease (KDAC wrist), as well as amyloid deposition diseases [14-16]. Traumatic causes include the rupture of the SLIL, usually resulting from falling on a hyperextended wrist.

In SNAC wrist, unlike SLAC, the SLI ligament is intact. The lesion results from nonunion or malunion of the scaphoid fracture [4]. Approximately 12% of scaphoid fractures fail to undergo osseous union ("nonunion") and require internal fixation [17]. Delayed diagnosis is associated with a nonunion rate of 88% [18]. It may remain asymptomatic and undiagnosed for long periods. If left untreated, gradual clinical and structural deterioration occurs due to progressive peri-scaphoid ligament injury, articular surface damage, and secondary wrist osteoarthritis [17].

Both lesions lead to abnormal wrist kinematics, resulting in dorsal intercalated segment instability (DISI) deformity over time. DISI progresses to degenerative arthritis of the radioscaphoid articulation, carpal collapse, and midcarpal arthritis [19]. The rate of progression depends on the load applied and the time elapsed from the injury.

Staging

Watson and Ballet [1] originally described the SLAC wrist in three stages (Table 1). In stage I, degenerative arthritic changes are limited to the tip of the radial styloid and the distal pole of the scaphoid. Stage II involves the entire radioscaphoid joint, and in stage III, degeneration progresses to the capitolunate joint. They noted that the radiolunate joint was rarely involved. Peterson B and Szabo RM [20] and Weiss KE and Rodner CM [2] added stage IV to include pancarpal arthritis, since they believed the radiolunate joint became involved in advanced disease.

**Table 1:** Stages of SLAC wrist according to Watson HK and Ballet F. Table was created using data from original article [1]. SLAC: Scapholunate advanced collapse.

Classification of SLAC			
Stage I	Stage II	Stage III	Stage IV
Radial styloid tip - distal scaphoid pole	Stage I + Radioscaphoid articulation	Stage II + Capitolunate articulation	Stage III + radiolunate articulation (pancarpal arthritis)

Vender MI et al. [3] originally classified SNAC into three stages (Table 2). Stage I involves the radial styloid and the distal scaphoid fragment. Stage II involves the proximal scaphoid and the capitate, and in stage III, the radioscaphoid, scaphocapitate, and capitolunate joints are affected [21].

**Table 2:** Stages of SNAC wrist according to Vender MI and others. Table was created using data from original article [3]. SNAC: Scaphoid nonunion advanced collapse.

Classification of SNAC		
Stage I	Stage II	Stage III
Radial styloid - distal scaphoid fragment	Proximal scaphoid - capitate (scaphocapitate articulation)	Radioscaphoid articulation + Scaphocapitate articulation + Capitolunate articulation

**Figure 2:**
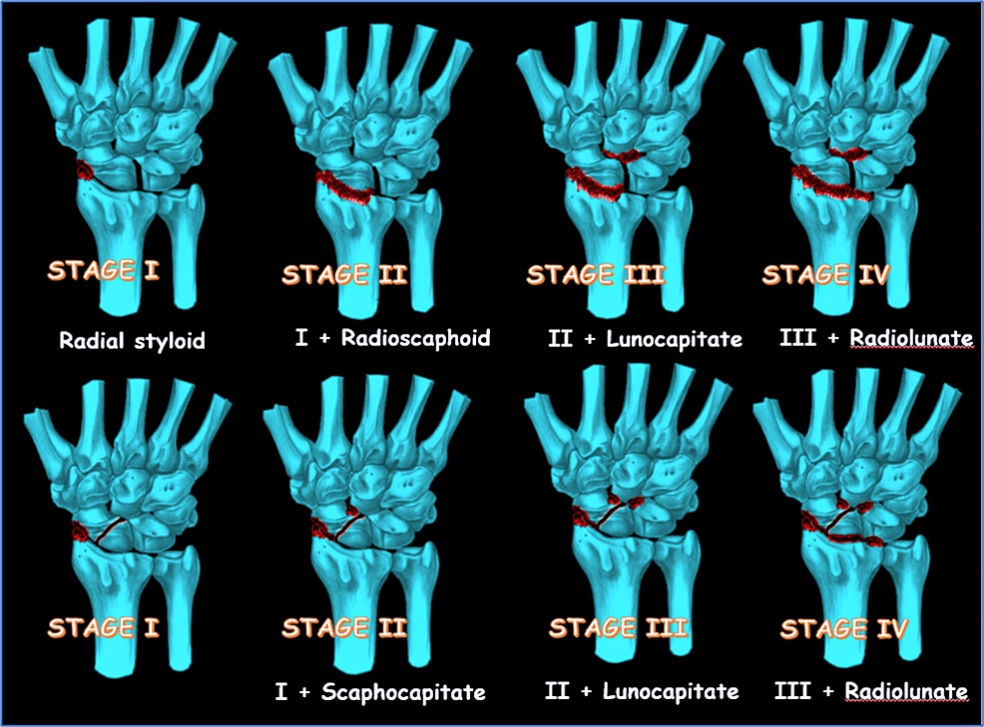
SLAC and SNAC staging. Source: Dr. Emmanouil Apergis. SNAC: Scaphoid nonunion advanced collapse; SLAC: Scapholunate advanced collapse.

The staging for SLAC and SNAC is shown in Figure 2.

Diagnosis

**Figure 3:**
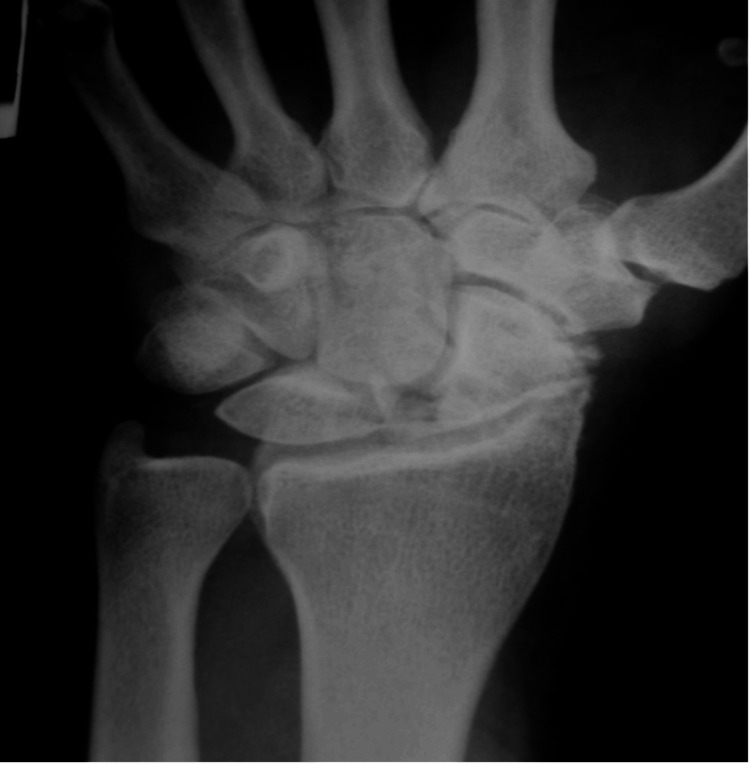
Wrist radiograph showing sclerosis and narrowing of the radioscaphoid articulation, with widening of the scapholunate (SL) interval - Stage II SLAC. Source: Dr. Emmanouil Apergis. SLAC: Scapholunate advanced collapse.

Both entities can remain asymptomatic for many years and go undiagnosed. Diagnosis is made through history, physical examination, and radiographs comparing both wrists. The patient presents with wrist pain, limited range of motion, and dorsal swelling, mainly in the scaphoid region [22]. Since no specific clinical test exists, imaging is vital for diagnosis [21]. In plain radiographs, the SLIL tear is diagnosed with widening of the scapholunate interval [23] (Figure 3), DISI malalignment, and narrowing of joint spaces.

Other imaging modalities include CT and MRI, which help diagnose SLAC and monitor its progression [5]. Li AE et al. [24] supported that MRI was 12 times more likely to show moderate osteoarthritis at the RL joint in SLAC wrists than radiographs. Luchetti R [25] supported the idea that diagnostic arthroscopy is essential for staging and surgical decision-making regarding the type of treatment. In each case, the choice of procedure should be made intraoperatively [26].

Treatment

Asymptomatic SLAC/SNAC wrists do not require treatment [5]. In symptomatic cases, the initial treatment approach should be conservative [27]. Nonoperative treatment includes immobilization with a splint, non-steroidal anti-inflammatory drugs (NSAIDs), and intraarticular corticosteroid injections. When these fail, various operative techniques have been described [5].

The progressive pattern of degeneration has not been effectively stopped by any procedure or medication in literature at this time [28]. Once arthritis (SLAC-SNAC wrist) has been diagnosed, regardless of the stage, the conventional surgical treatment options are traditionally limited to two motion-preserving salvage procedures that change the mechanics of the joint: proximal row carpectomy (PRC) and four-corner fusion (4-CF).

Operative treatment varies and depends on the stage of the lesion. For early stages, less definitive procedures with preservation of the scaphoid are sometimes used to delay more definitive surgery (PRC, 4-CF), such as arthroscopic resection arthroplasty of the styloscaphoid joint [29] or resection and interposition arthroplasty of the styloscaphoid joint combined with dorsal capsulodesis of the scaphoid [28]. In addition, arthroscopic treatment of SLAC wrist I and II by large styloidectomy associated with a tendon autograft interposition arthroplasty tightened as a hammock has been described [30]. Moreover, classical methods like PRC, arthrodeses, capsulodeses, and tenodeses have also been described in the literature.

Proximal Row Carpectomy

PRC is a motion-preserving surgical procedure for treating degenerative wrist conditions. Although it is generally considered a salvage procedure and significantly alters wrist biomechanics [31-35], it has been shown to reliably reduce pain and improve function [36].

PRC involves the resection of the proximal carpal row, including the triquetrum, lunate, and scaphoid bones, allowing the capitate to articulate with the lunate facet of the distal radius. The creation of the new articulation between the radius and the capitate maintains wrist motion and can relieve pain. The main indication for this procedure is the preservation of the articular surfaces of the lunate fossa and the head of the capitate (stage II SLAC/SNAC). Typically, the wrist is immobilized for 3-4 weeks post-operatively [28].

PRC variants are primarily used in stage III SLAC and SNAC lesions. They include interposition arthroplasty [37] and chondroplasty [38].

Arthrodeses

Four-Corner Fusion (4-CF)

**Figure 4:**
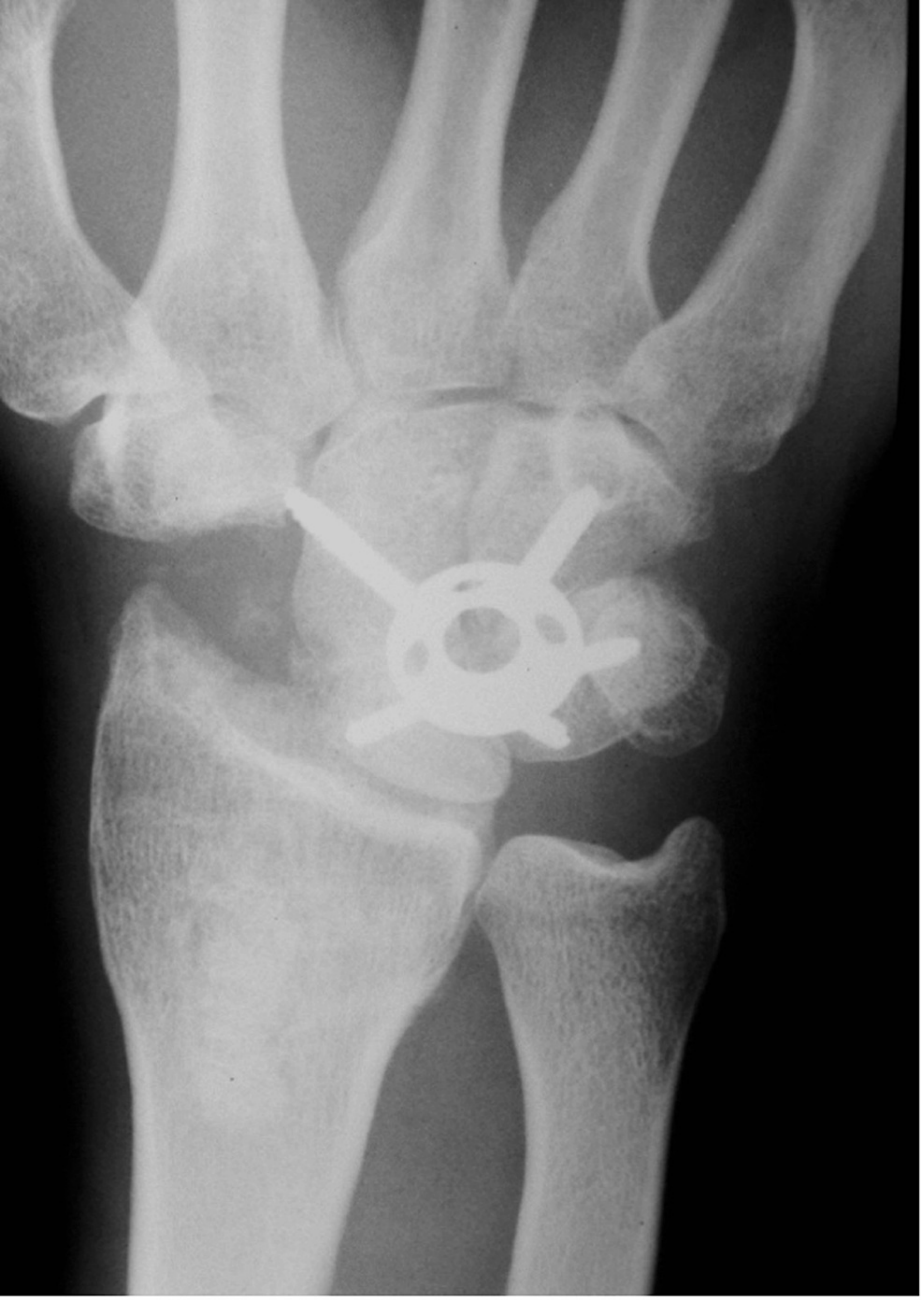
Four-corner fusion and scaphoid excision. Source: Dr. Emmanouil Apergis.

Watson HK and Ballet FL first described 4-CF in 1984 for treating SLAC and SNAC wrist [1]. They described scaphoid excision and fusion of the lunate, capitate, hamate, and triquetrum bones with K-wires. Later, fixation with screws [39] and staples [40] was described, with successful results. Another option for fusion was the circular plate (Figure 4) (locking, non-locking, and radiolucent locking plates), introduced for more stable fixation to allow the patient to start early rehabilitation [41-43]. However, many complications have been described in the literature, such as hardware breakage, irritation, and dorsal impingement [44-47], with no significant differences between methods. While there are some important differences in the range of motion and grip strength, these differences are unlikely to be clinically relevant.

4-CF is usually performed through a dorsal approach. The scaphoid and the posterior interosseous nerve are resected, and the adjacent surfaces of the lunate, capitate, hamate, and triquetrum are decorticated. Then, they are reduced and fixed [48], making the radiolunate joint the load-bearing joint of the wrist. Immobilization of the wrist for 6 to 9 weeks is suggested [28].

According to Watson HK and Ballet FL [1], 4-CF is preferred in stage III SLAC since the proximal pole of the capitate is degenerated, and PRC is excluded as an option. Whether PRC or 4-CF has better outcomes for SLAC II wrists is still under consideration [49].

4-CF Variants

Three-corner fusion (3-CF): 3-CF is an alternative for SLAC and SNAC stages II and III. It involves the resection of the scaphoid and triquetrum and the fusion of the lunate, capitate, and hamate. The excision of the triquetrum improves ulnar deviation, prevents ulnocarpal impingement, and is also used as bone graft in the fusion [50]. Delattre O et al. [51] describe, in a 30-patient study, comparable results between 4-CF and 3-CF. It is a simpler procedure than 4-CF but more demanding than PRC. ElKaref E et al. [52] also report comparable results between 4-CF and 3-CF, with a slightly better range of motion in 4-CF and similar grip strength.

**Figure 5:**
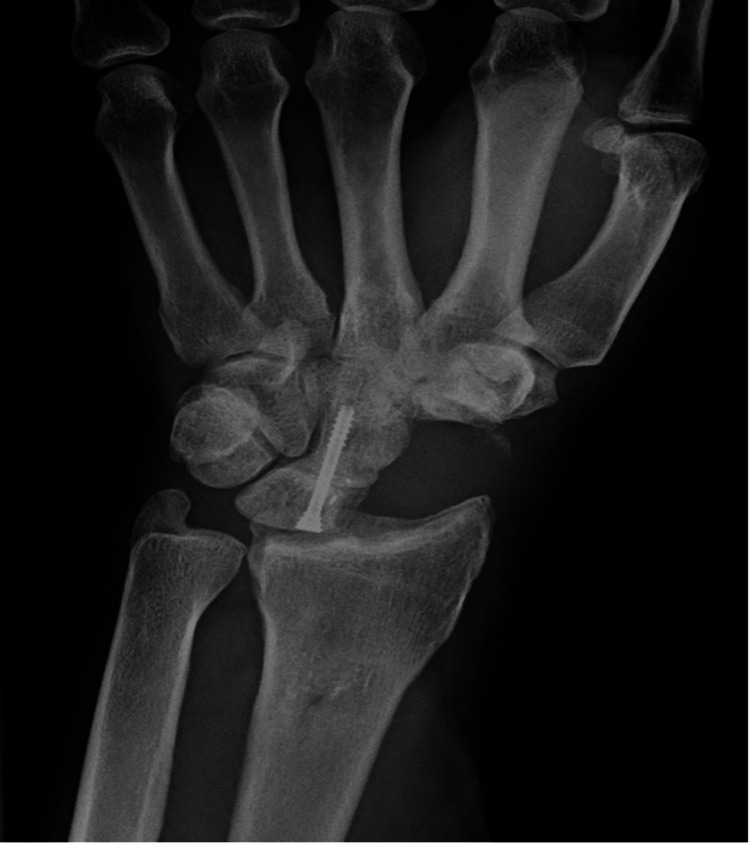
Capitolunate fusion and scaphoid excision. Source: Dr. Emmanouil Apergis.

Capitolunate fusion: Capitolunate arthrodesis was first described in 1966 by Graner O to treat Kienböck's disease (Figure 5) [53]. In 1984, Watson HK and Ballet FL proposed capitolunate arthrodesis as well as 4-CF for the treatment of SLAC wrists [1]. This procedure has been proposed as an alternative in the treatment of advanced SLAC/SNAC arthritis, as it preserves more range of motion compared to total wrist arthrodesis. It is combined with scaphoid excision, although some suggest triquetrum excision as well [54], since it decreases the chance of pisotriquetral arthritis [55]. Dunn JC et al. [55] describe in their late systematic review (2020), examining 80 patients with CLA, that 11% of patients showed complications (non-union, Herbert screws migration) and 14% had to be reoperated (screws removal, wrist arthrodesis, wrist arthroplasty). However, 96% of patients showed union rates. This review suggests that CLA is viable in SLAC and SNAC wrists with similar complication rates to the 4-CF [55].

PRC versus 4-CF

Extensive literature has compared the outcomes of 4-CF and PRC. Mulford JS et al. [56] suggest that both groups yield similar results in their systematic review regarding grip strength, range of motion (ROM), and subjective outcomes (pain relief). Although PRC had higher osteoarthritic changes post-operatively, most cases were asymptomatic. Conversely, 4-CF showed more complications (10% more), including non-union and complications related to hardware.

Saltzman BM et al. [57] conducted a systematic review in which 242 wrists were reviewed (from seven studies). They concluded that in 4-CF, the most common complication was non-union, and in PRC, synovitis causing edema. 4-CF showed better grip strength and radial deviation. PRC showed better range of motion (mostly in flexion-extension) and overall fewer complications.

Ahmadi AR et al. [49], in their more recent systematic review (2021), included 15 articles (322 4-CF, 328 PRC). In most of the reviewed studies, the patients had sustained stage II and stage III SLAC/SNAC lesions. They recommend PRC over 4-CF if there is no midcarpal osteoarthritis since it is a relatively simple procedure with a short learning curve. Additionally, PRC shows no hardware-related complications and provides similar long-term outcomes.

According to Garcia BN et al. [58], PRC and 4-CF demonstrated similarly low rates of conversion to total wrist arthrodesis. The rate of secondary surgical procedures, such as revision 4-CF, hardware removal, irrigation, and debridement, following 4-CF was significantly higher compared with PRC. Since both procedures have similar clinical outcomes, PRC may be a preferable treatment for stage-II SLAC/SNAC wrist arthritis.

Capsulodesis

Salvage procedures are not indicated in young and active patients in the early stages of SLAC (stages I & II). In these cases, dorsal capsulodesis, with or without scaphoidectomy, has been suggested as an alternative to restore more alignment of the scaphoid by tightening the dorsal ligaments [28] (secondary stabilizers of the scapholunate joint) [59]. Trumble TE et al. [60] described scaphoidectomy combined with dorsal capsulorrhaphy for stage II SLAC and SNAC lesions. Although in a limited series of 8 patients, it is the only capsulorrhaphy with isolated scaphoidectomy procedure without interposition materials.

It is mainly indicated in elderly patients with advanced wrist arthritis since it provides pain relief while being a less invasive procedure. The main concern is the expected progressive collapse of the wrist and the long-term outcomes that should be reviewed with a larger series [60].

Tenodesis

Midcarpal tenodesis for arthritis stage II SLAC or SNAC has been proposed: a) As a surgical choice, when lunocapitate and radiolunate joints are intact; (b) As a method that, in case of failure, allows for the possibility to carry out one of the classical salvage operations (PRC or four-corner arthrodesis with scaphoidectomy); (c) With the expectation that midcarpal tenodesis will keep, in the long term, the lunocapitate joint aligned; and (d) For surgeons who dispute the necessity of fusing normal (non-arthritic) joints (four-corner arthrodesis) or the creation of asymmetric joints (joint between radius and capitate in PRC) or dislike removing bones with normal cartilage (lunate-triquetrum) in PRC.

Scaphoidectomy and midcarpal tenodesis have been used in the past for stage II SLAC/SNAC wrist arthritis with partial flexor carpi radialis transfer [25]. The method provides good clinical but poor radiological results.

Luchetti R et al. [25] suggest midcarpal tenodesis with flexor carpi radialis (FCR) combined with scaphoid excision. This technique was proposed as an alternative when the midcarpal and the radiolunate joints are completely normal (SLAC and SNAC Stage II). He reports 14 cases out of 18 operated by him for stage II SLAC and SNAC with a 7-year follow-up, showing poor radiological results (progressive joint degeneration of carpal bones, reduction of carpal height, increase of DISI deformity, LT ulnar translation, capitate radial, and proximal progression) despite good clinical results. Filho G et al. [61] propose for stage II SNAC lesions resection of the distal pole of the scaphoid combined with proximal tenodesis with extensor carpi radialis brevis (ECRB). Although the patient number is small (six patients), they present this technique as a safe alternative with few complications.

Discussion

SLAC and SNAC lesions are the two most common causes of post-traumatic wrist arthritis. This article reviews the etiology, staging, diagnosis, and management of these lesions, particularly of stage II. Prompt diagnosis is critical as it affects the management and prognosis.

Many procedures have been described for the operative management of stage II lesions, with the most popular being proximal row carpectomy (PRC) and four-corner fusion (4CF). Variants of these procedures have also been proposed as alternatives, such as interposition arthroplasty and chondroplasty, three-corner fusion (3CF), capitolunate arthrodesis (CLA), capsulodesis, and tenodesis.

PRC is generally considered a salvage procedure, as it significantly alters wrist biomechanics [31-35]. It has been shown to reliably reduce pain and improve function [36]. The main prerequisite for this procedure is the preservation of the articular surfaces of the lunate fossa and the head of the capitate (stage II SLAC/SNAC).

4-CF is mainly preferred in stage III SLAC, according to Watson [1], since the proximal pole of the capitate is degenerated, and PRC is excluded as an option. More recent studies suggest PRC over 4-CF if there is no midcarpal osteoarthritis since it is a relatively simple procedure with a short learning curve. In addition, PRC shows no hardware-related complications and provides similar long-term outcomes (Ahmadi AR et al. [49]). Other studies concluded that the most common complication in 4-CF was non-union, and in PRC, synovitis causing edema. A recent study by Garcia BN et al. [58] suggested that since both procedures have similar clinical outcomes, PRC may be preferable for stage II SLAC/SNAC wrist arthritis.

Alternative bony procedures for stage II SLAC/SNAC wrists include 3-CF and Capitolunate arthrodesis (CLA). Recent studies regarding 3-CF show comparable results with 4-CF, with a slightly better range of motion (ROM) in 4-CF and similar grip strength. It is a simpler procedure than 4-CF but more demanding than PRC [51]. Capitolunate arthrodesis shows very low complication rates (approximately 10%), primarily non-union and screw migration, but it has been suggested as having similar complication rates to 4-CF [55].

Finally, soft tissue operations such as capsulodesis and tenodesis have been described. Dorsal capsulodesis is a better option for the elderly with advanced wrist degeneration since it is less invasive and provides pain relief [60]. Tenodesis, on the other hand, has been proposed in younger patients to avoid early salvage procedures. Various tendons have been used for this kind of operation (FCR, ECRB) in a small number of patients with good clinical outcomes despite poor radiological results [61].

## Conclusions

SLAC and SNAC are the most common causes of wrist arthritis. Treatment varies according to the stage. Various alternatives for stage II SLAC and SNAC have been proposed, such as PRC, 4-CF, 3-CF, capitolunate fusion, capsulodesis, and tenodesis. Partial arthrodeses (4-CF, 3-CF, CLF) appear to yield similar results. Soft tissue procedures are preferred in younger patients to avoid early salvage procedures and preserve the ROM.
